# Unpacking the Gateway Hypothesis of E-Cigarette Use: The Need for Triangulation of Individual- and Population-Level Data

**DOI:** 10.1093/ntr/ntac035

**Published:** 2022-02-09

**Authors:** Lion Shahab, Jamie Brown, Lies Boelen, Emma Beard, Robert West, Marcus R Munafò

**Affiliations:** Department of Behavioural Science and Health, University College London, London, UK; SPECTRUM Consortium, Edinburgh, UK; Department of Behavioural Science and Health, University College London, London, UK; SPECTRUM Consortium, Edinburgh, UK; Sandtable Ltd, London, UK; Department of Behavioural Science and Health, University College London, London, UK; SPECTRUM Consortium, Edinburgh, UK; Department of Behavioural Science and Health, University College London, London, UK; SPECTRUM Consortium, Edinburgh, UK; School of Psychological Science, University of Bristol, Bristol, UK; MRC Integrative Epidemiology Unit, University of Bristol, Bristol, UK

As recent publications in *N&TR* highlight,^1^ there is considerable debate whether e-cigarettes act as a causal gateway to subsequent smoking in adolescents. Answering this question requires a clear specification of what we mean by “gateway” in this context. Unlike the original hypothesis, which postulated a causal progression from legal/“soft” (eg, alcohol) to illicit/“hard” (eg, heroin) polydrug use, initiated in adolescence,^2^ current gateway discussions in tobacco control center around the idea of what is essentially a transition from less (e-cigarette) to more harmful (cigarette) modalities of nicotine use.

This complicates causal inference; for the original hypothesis it was possible to assess changes in sensitivity to, and rewarding effects of, a destination drug following exposure to a gateway drug in animal models, but such highly controlled experimental work in model systems is impossible when both the putative gateway and destination drug are the same. Randomized controlled trials are equally unfeasible: it would be unethical to randomize adolescents to try or not try e-cigarettes and impractical to assess relatively long-term outcomes (ie, transition to smoking, potentially several years later). We therefore must rely on observational data. Here, we introduce the idea of a triangulation framework, using evidence from different approaches to provide robust answers to causal questions, and apply this to the problem of the gateway to assess the current balance of evidence.

Triangulation attempts to support stronger causal inference by considering findings across multiple methodological approaches, each with different sources of bias.^3^ For research into gateway effects of e-cigarette use, we define two major categories of approach: (1) individual-level approaches (ie, where individual participants provide data), and (2) population-level approaches (ie, using summary data from whole populations). Cutting across these, one can also distinguish between cross-sectional comparisons between individuals or populations, and longitudinal comparisons of individuals or populations over time.

At the individual level, numerous cross-sectional and prospective studies show a strong positive association between e-cigarette use and smoking cigarettes.^4^ Young people who report using e-cigarettes are more likely to report smoking, both concurrently and in the future. However, these studies are potentially subject to measurement error (eg, misreporting of smoking status, especially among youth^5^) and confounding.^6^ Just because e-cigarette use precedes cigarette use does not mean that e-cigarette use *caused* subsequent smoking: adolescents that try e-cigarettes may have tried cigarettes anyway due to some underlying common liability, such as a genetic predisposition to risky behavior.^7^ Although it is possible to reduce such confounding statistically (eg, propensity score matching),^8^ success depends on all relevant confounders being included (which is unlikely, resulting in unmeasured confounding), included confounders being measured accurately (again unlikely, resulting in residual confounding), and the association with the outcome being modeled through an appropriate function (eg, exponential). Even small model misspecifications can result in spurious associations.^6^ No matter how many studies one conducts with the same sources of bias, the limitations of this approach remain, running the unintentional risk of creating the appearance of robustness and acceptance that causal claims are true.^9^

What is needed then are complementary and independent lines of evidence that address this problem of confounding by combining methodologies that have distinct potential biases. If results across approaches align, this provides greater confidence in a causal interpretation, since it is unlikely that different sources of bias would conspire to give the same result in each case. There are numerous methods that can be applied to individual-level data (Supplementary Table 1).^3^ One of these is the use of “negative control” outcomes; these exhibit a similar confounding structure to smoking, but have no plausible mechanism through which e-cigarette use could influence them (eg, unprotected sexual intercourse). If e-cigarette use shows a similar association with this as with smoking, it suggests that confounding (eg, via risk taking personality) is the most likely culprit rather than there being a true causal association.^10^ Indeed, there are studies showing associations with such outcomes, including illicit prescription medication use.^11^

Instrumental variable analysis constitutes another individual-level approach. This would require an instrument causally related to e-cigarette use but without any plausible causal connection with smoking. Such studies could exploit environmental instruments or use genetic variants as proxies for the exposure (as in Mendelian Randomisation, MR).^12^ One environmental instrument could be access to e-cigarettes, for example by density of vape shops in the locality, opportunity for online purchasing, or pricing of e-cigarettes. Thus, if it turns out that adolescents with greater access specifically to e-cigarettes are more likely to smoke, this would support a causal role for e-cigarettes in the take-up of smoking. Identifying genetic instruments that differentiate e-cigarette from cigarette use may be more difficult. Early evidence indicates a common genetic vulnerability to both smoking and e-cigarette use, which may reflect a broad risk taking phenotype (in itself suggestive that at least part of the association between e-cigarette use and smoking may be noncausal).^13^ Other genetic approaches beyond MR, including twin and sibship comparison studies,^14^ may hold promise for disentangling genetic confounders and intergenerational transmission to assess true causal associations between e-cigarette use and smoking.

Turning to the population level (Supplementary Table 1), time series analyses can examine associations over time between prevalence of e-cigarette and cigarette use in potentially vulnerable age groups. Because the whole population is being studied, this rules out individual factors accounting for any association found. Population-level studies can be biased by confounding variables that operate at the population level, but these will not overlap with individual confounders. For example, a country might relax or tighten regulations for e-cigarettes and cigarettes at the same time or fund a media campaign targeting both types of product. Therefore, it remains important to adjust for population-level confounding as far as possible (eg, shifts in policy). Population-level time series analyses have the additional advantage of directly estimating the population-level effect of e-cigarettes as has already been done for the impact of e-cigarette use on smoking cessation.^15^ Importantly, such analysis should include appropriately lagged effects to assess any putative gateway.

Other methodological approaches at population level include cross-context comparisons and natural experiments, which can exploit the likelihood that confounding structures will be different across populations in dissimilar contexts (either historically, eg, because of differently patterned behaviors, or by design, eg, due to legislative changes introduced in one but not in another context).^3^ Similar findings across contexts could not be explained readily by confounding, while different results would be unlikely due to true differences in causal effects between populations. No studies of this kind have been reported. A preliminary look at population data shows that over the same time-period that e-cigarette use increased, cigarette use decreased, and this is found in markedly different contexts,^16,17^ suggesting a limited role of confounding.

One can also use modeling (Supplementary Table 1) to assess the extent to which individual- and population-level estimates of gateway effect agree with each other, for example, with microsimulation or agent-based models, employed for now-or forecasting in economics, and increasingly applied to guide health policy.^18^ Our group has developed such a model in collaboration with Sandtable, a data analysis company. Here, adolescent cigarette uptake is calibrated to match rates of decline prior to e-cigarettes becoming popular, e-cigarette uptake is matched to observed values and the model run to estimate smoking prevalence in counterfactual scenarios, using different associations between e-cigarette and cigarette use. We find that the gateway effect estimated from individual-level studies^19^ predicted far less of a reduction in population smoking prevalence in the relevant age group than was actually observed (Figure 1), making it unlikely that individual-level effects were genuine.

**Figure 1.**
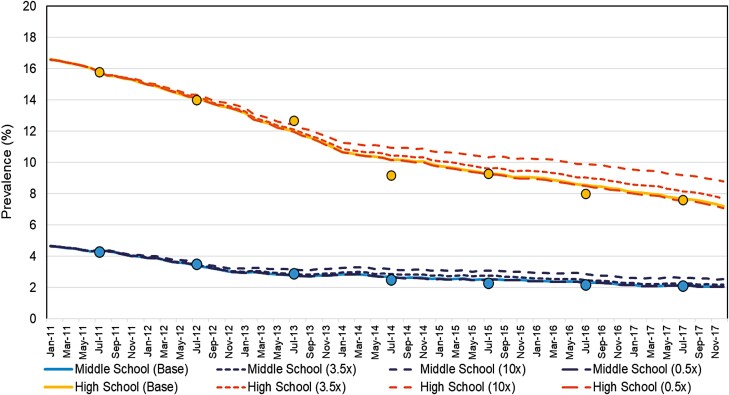
Observed^a^ and modeled past 30-day youth smoking prevalence in the United States 2011–2017, using the youth e-cigarette microsimulation model^b^. ^a^Observed values (filled circles) come from the National Youth Tobacco survey 2011–2017. ^b^The model, data, and description can be found online (https://osf.io/pycqj/); briefly, the microsimulation consists of 50 000 agents, each of which represents an individual as defined by the characteristics relevant for the question (ie, age, smoking status, vaping status), who at monthly intervals decide to take up smoking and/or vaping; the probabilities that govern these decisions are determined by the user (ie, a multiplier that adjusts the probability of smoking uptake for agents that already vape and vice versa). Different postulated effect sizes (multipliers) for the strength of association of e-cigarette use with uptake of cigarettes is provided as odds ratios in brackets (x); the base model assumes no effect of e-cigarettes (solid line), broken lines indicate either a postulated positive (OR > 1) or negative association (OR < 1) between e-cigarette use and smoking uptake.

Lastly, while we have focused on the analysis of individual- and population-level aggregated data, with the proliferation of mobile phone technologies, *n*-of-1 studies may also offer an insight into possible gateway effects (eg, by following transitions of individuals in different contexts using ecological momentary assessment). Further, most designs and statistical methods mentioned here rely heavily on Frequentist hypothesis testing, which can lead to nonsignificant findings being conflated with evidence of no effects. One remedy is the use of Bayes factors which assess the extent of evidence for the null hypothesis and can also determine if the data analysis is insensitive or underpowered.^20^ Many of the approaches mentioned in Supplementary Table 1 have Bayesian equivalents, which in themselves can offer a method of triangulation, as prior knowledge from already published studies can be incorporated.

If we carry on as we are, we are unlikely to address this important scientific and public health question satisfactorily and may merely perpetuate disagreement by selective reporting of results that favor one direction or the other of a gateway effect. In addition to encouraging single method investigations using new and diverse methodologies, we would therefore argue for a common, transparent approach that involves prospective registration of a triangulation framework based on individual- and population-level data and a priori specification of what would be considered sufficient evidence in each direction of the postulated gateway, based not only on statistical significance but also on clinically or theoretically meaningful effect sizes (for an example see https://osf.io/nd2qk). Taking a collaborative approach, involving groups with different prior beliefs, would not only increase feasibility of triangulation but also help unify our field. Based on the current balance of evidence, using triangulated data from recent population-level cross-contextual comparisons, individual-level genetic analyses and modeling, we do believe, however, that causal claims about a strong gateway effect from e-cigarettes to smoking are unlikely to hold, while it remains too early to preclude other smaller or opposing effects.

## Supplementary Material

A Contributorship Form detailing each author’s specific involvement with this content, as well as any supplementary data, are available online at https://academic.oup.com/ntr.

ntac035_suppl_Supplementary_Data

ntac035_suppl_Supplementary_Table_S1
