# Supplementary material for: Unpacking the Gateway Hypothesis of E-Cigarette Use: The Need for Triangulation of Individual- and Population-Level Data
Source: Nicotine Tob Res. 2022 Feb 9;24(8):1315–8. doi: 10.1093/ntr/ntac035 (PMC9278819; doi:10.1093/ntr/ntac035)
Supplement: ntac035_suppl_Supplementary_Table_S1 [file ntac035_suppl_supplementary_table_s1.docx]

**Supplementary Table 1: Examples of different individual- and population-level approaches to triangulation in e-cigarette gateway research**

|  | ***Design*** | ***Description*** | ***Examples*** | ***Key biases*** |
| --- | --- | --- | --- | --- |
| ***Individual-level data*** | | | | |
| Standard regression-based/multilevel/fixed effect models | Cross-sectional; longitudinal | Exposure outcome association is measured, adjusted for confounding variables. For longitudinal designs correlation among repeated measures is considered. | Multivariable logistic regression: longitudinal survey to assess association between baseline exposure to e-cigarette use among never smokers and follow-up smoking rates using multilevel models, generalized estimation equations and others. | Residual and unmeasured confounding of individual-level influences; reverse causality/misreporting (cross-sectional); systematic attrition (longitudinal). |
| Propensity-score matching (PSM) based studies and inverse probability treatment weighting (IPTW) | Cross-sectional; longitudinal | Exposure outcome association is measured, comparing outcome in exposed and non-exposed individuals who are matched on basis of maximised similarity in propensity score (computed from likely confounders of exposure-outcome association, with confounders, exposure and outcome assessed retrospectively/concurrently in cross-sectional design or prospectively in longitudinal design).  Instead of matching on the propensity score weighting (IPTW) on the propensity score can also be used. Unlike matching, weighting keeps most observations in the analysis and can therefore  offer increased precision when estimating effects. A weighted outcome regression model can be use with treatment status as the independent variable to derive adjusted treatment effect estimates, as covariates are expected to be balanced in the weighted population. To account for the fact that the pseudo-population size is inflated or deflated relative to the original study population and that weights are estimated, a robust, sandwich type estimator can be used. | Prospective propensity score matching: longitudinal survey of tobacco-naïve individuals at Time 1, when confounders are assessed, followed up to assess e-cigarette use at Time 2, when e-cigarette initiators are matched to non-initiators basis on propensity score computed from Time 1 confounders, followed up to assess cigarette use at Time 3 in e-cigarette initiators and matched non-initiators. | Residual and unmeasured confounding of individual-level influences; misreporting (cross-sectional); systematic attrition (longitudinal); applying PSM may result in a small sample size because unmatched patients are dropped from the final sample. |
| Structural equation modeling | Longitudinal | Repeated measures of exposure and outcome are used to account for the possibility of an association between the exposure and the unexplained variability/unmeasured confounding in the outcome; adjusting for both time-invariant and time-variant confounders. | Parallel process latent growth curve analysis: longitudinal survey to assess association of e-cigarette imitation with smoking initiation across time with concurrent modelling of inverse effect (smoking initiation to e-cigarette initiation), creating latent variables for e-cigarette initiation, cigarette initiation and controlling for observed confounders. | Residual confounding and unmeasured fixed confounding of individual-level influences. |
| Case-control studies with different confounding structures | Cross-sectional | In a case-control study, participants who report smoking are compared to those who do not report smoking in terms of retrospective e-cigarette use. An extension of this involves using multiple control groups with different confounding structures. If the sources of bias in the different groups are indeed different, this would produce different associations, whereas a causal effect would produce the same observed association. | For example, one could compare the association between e-cigarettes use and cigarette use in different cohorts e.g. those surveyed between 2010 and 2015 and those surveyed between 2015 and 2020 or those surveyed in the north of England and those surveyed in the south of England. Both would have different confounding structures. | Recall bias, sampling bias; only one outcome considered; not useful for rare risk factors. |
| Twin studies | Cross-sectional, longitudinal | Twin studies provide a basis for exploring the importance of risk factors on an outcome by controlling the genetic variations. Monozygotic (identical) twins are expected to share all of their genes, whereas dizygotic (fraternal) twins share only about 50% of them. Twin pairs, having been reared in the same household, are expected to be highly concordant for environmental experiences. | a shared genetic  vulnerability to develop dependence on a range of  different drugs.  Twin studies can be used to assess if there is a genetic vulnerability to developing dependence on e-cigarettes and cigarettes. | Results from twin studies cannot be directly generalized to the general population; unmeasured confounding (biased by the influence of the non-shared environment). |
| Instrumental variable analysis | Cross-sectional or longitudinal | Instrumental variables are variables that are robustly associated with an exposure of interest, but are not associated with the outcome (other than through the exposure) or with confounders of the exposure-outcome association. Mendelian randomization (MR) extends this approach by using a genetic variant as a proxy for the exposure. | For example, one could use attitudes or view of the harmfulness of e-cigarettes as an instrumental variable in testing the association between e-cigarette use and uptake of smoking. | Weak associations between the instrument and the exposure can introduce bias; exclusion restriction criterion violation is a main source of bias in MR (due to horizontal pleiotropy); population stratification can also be a source of bias in MR, which may require focusing on an ethnically homogeneous population. |
| Positive/negative control studies* | Cross-sectional; longitudinal | A negative control outcome acts as a surrogate for the actual outcome - the negative control should be subject to the same potential sources of bias as the outcome but is not caused by the exposure of interest. A positive control outcome is an outcome that is known to be causally related to the exposure. It can be used to ensure the population sampled generates associations that would be expected. | For example, we could assess if an association exists between youth use of e-cigarettes and attitudes towards cigarettes (positive control outcome). | Differences in biases of real and negative/positive control exposure/outcome; unknown causal effect of negative control; no causal effect of positive control. |
| Regression discontinuity analysis | Repeated cross-sectional | Regression discontinuity involves using a continuously measured variable to assign an exposure to individuals based on a threshold rule. Individuals just above the threshold are expected to be similar in their distribution of covariates to individuals just below the threshold, resulting in exchangeability. | A threshold could be the date of the introduction of e-cigarettes into the market and the forcing variable used is the time of the survey/interview. If the interview date of the survey is after the introduction of e-cigarettes, individuals are classed as treated, while those interviewed before are in the control group. | Assumption that there is a sharp cut-off, around which there is a discontinuity in the probability of assignment from 0 to 1; estimates will be biased if cutoffs are not strictly implemented; model misspecification (continuous variable may not have a linear association). |
| Difference-in-differences | Repeat cross-sectional | Difference in differences assesses the differential effect of a treatment on a 'treatment group' versus a 'control group' in a natural experiment. It does this by calculating the effect of a treatment on an outcome by comparing the average change over time in the outcome variable for the treatment group, compared to the average change over time for the control group (generally using an interaction/or moderation term). | This is most useful when one geographical area receives a treatment, and another does not. For example, one could assess changes over time in a control group not exposed to changes in e-cigarette policy versus an experimental group where e-cigarette have become more freely available. | Does not account for unobservable time-varying variables; non-equivalence of study groups (other than exposure); misreporting. |
| Dynamic prediction with time-varying covariates in survival analysis: cox regression, joint models and landmark analysis | Longitudinal | Time-varying survival models (e.g., Cox regression model) can be used to assess the association between an observed longitudinal measure and the hazard of an event. However, this approach fails to account for measurement error in the covariates. Joint models address this issue by modelling simultaneously the profile of the covariate and the time-to-event data. Within this approach, a linear mixed model for the underlying longitudinal trajectories of the covariate is linked to the survival model using shared random effects. This approach allows inference on the association between the hazards of an event and the longitudinal covariate accounting for random measurement error. Another way to deal with time-varying covariates involves selecting a small number of index time points. Survival analysis is then done on the subjects who remain event-free at the specified index times and follow up beyond the index times. Thus, the baseline time for each analysis changes. | In a joint model framework, a linear mixed model for the underlying longitudinal trajectories of self-reported e-cigarette (time-varying covariate) use could be linked to time-to-event (survival data) in the form of self-reported smoking using shared random effects. | Residual and unmeasured confounding of individual-level influences; joint models are parametric and have stronger assumptions than some semi-parametric survival models; for landmark analysis you can’t directly address how associations vary over and time points selected are arbitrary and open to bias. |
| Micro-simulation models | N/A | Simulation models which mimic the operation of government programs and demographic processes from the bottom-up, based on individual ("micro") members of a population moving between states in a stochastic manner, depending on key parameters set at the individual level. This can include interactions between agents (e.g., in agent-based models) to generate emerging macro-level observations. | A microsimulation model calibrated on data from a population level survey that assigns different individual transition probabilities from not smoking to smoking based on variety of individual level characteristics, including e-cigarette use, to forecast changes in smoking prevalence among young adults and compare with observed smoking rates. The postulated impact of e-cigarette use on individual state transitions can be varied to provide best fit to data. | Model misspecification (relevant confounders not included), results being only as good as the parameter estimates used and inputs included. |
| Multi-state Markov model | Longitudinal | The multi-state Markov model is a useful way of describing a process in which an individual  moves through a series of states in continuous time. These models can provide an insight into the effects of exposures on each state. | A multi-state Markov model could be developed based on smoking stage (e.g., non-smoker, experimentation, regular smoker). A scenario could compare a simulated cohort where all participants take up e-cigarettes at the start to a cohort where all remain non-e cigarette users. If there is a gateway, we may expect e-cigarette users to have greater progression across the smoking stages. | Model misspecification (relevant confounders not included), results being only as good as the parameter estimates used and inputs included. |
| ***Population-level data*** | | |  |  |
| Time series trend analysis | Repeat cross-sectional | A trend analysis fits a trend (e.g. linear, logarithmic or polynomial) to various time series. Comparisons can then be made to assess if the trajectory for two series mirror each other. Segmented trends may also be fitted, where the relationship between the outcome and the predictor variables are piecewise linear, to assess if changes in the trends occur for the two series at a similar time. Additional variables can be added to adjust for confounding. Although traditional GLM models can be used, more complex models such as GAMM and GLS allow for the inclusion of autocorrelation terms. GAMM also includes smoothing terms to account for seasonality. | Comparisons can be made to assess if the trajectory in growth of electronic cigarettes among young adults mirrors a trajectory in use of cigarettes among young adults. One could also assess if the trajectory in cigarette use in a country with a liberal approach to e-cigarettes is similar to a country where e-cigarettes are banned or highly regulated. | Model misspecification, unmeasured confounding at population level. |
| Multiple time series analysis | Repeat cross-sectional | In multiple time series analysis, the extent to which two series covary with each other over time is assessed. Additional variables can be added to adjust for confounding. Multiple time series analysis often involves complex models which account for seasonality and autocorrelation and remove underlying trends (e.g., ARIMAX, SVAR). | Assess whether changes in the prevalence of use of e-cigarettes is among young adults is associated with changes in the prevalence of use of cigarettes by young adults. | Model misspecification, unmeasured confounding at population level. |
| Interrupted Time-series analysis | Repeat cross-sectional | An interrupted time series, similar to a segmented regression, can be used to assess if the average of a time series changes abruptly but temporarily (pulse effect) following the introduction of a policy or changes abruptly but is maintained (step level change) following the introduction of a policy. Additional variables can be added to adjust for confounding. Interrupted time series analysis often involves complex models which account for seasonality and autocorrelation and remove underlying trends (e.g. ARIMA).  An alternative design is the controlled ITS which involves adding a control series, which was not exposed to the intervention, to the basic ITS design. One could also make adjustments using a control group to the output time series by subtracting the values of one from the other, or use data-driven techniques such as synthetic controls that derives a comparison unit from a weighted average of eligible comparison units (the ‘donor pool’) that minimizes the differences between pre-intervention trends in the treated and synthetic control series. | Assess wherther there is a change in the prevalence of youth smoking before and after the introduction of e-cigarettes into the UK market. | Model misspecification; unmeasured confounding at population level. |
| Time series forecasting | Repeat cross section | Comparisons can be made between the true time series and a forecasted time series using data prior to some policy implementation or event. If the two diverge substantially, this may indicate an influence of the policy implementation or event. Additional variables can be added to adjust for confounding. This involves complex models which account for seasonality and autocorrelation and remove underlying trends (e.g., ARIMA). | Forecast using data prior to the introduction of e-cigarettes youth smoking and compare this to the actual prevalence of youth smoking over time. If the actual prevalence is higher than the forecasted prevalence this might suggest a gateway effect. | Model misspecification; unmeasured confounding at population level. |
| Natural experiment | Repeat cross-sectional | Several statistical methodologies outlined above can be applied here, but the main drive is that differences in a desired outcome are compared for populations that are exposed to variations introduced in the environment, but which have otherwise a similar confounding structure. | Compare changes in youth smoking rates over time in countries where a ban on e-cigarettes is introduced compared with changes in countries with liberal access to e-cigarettes. | Unmeasured confounding resulting in non-equivalent populations; no proper segmentation of exposure (e.g., policy violations); potential selection bias; measurement differences across populations. |
| Cross-context comparisons | Cross-sectional | Several statistical methodologies outlined above can be applied here, but the main drive is that outcomes are compared in populations from different contexts/with inherent difference (e.g. in population make-up), resulting in differing confounding structures. | Compare the sex-specific association of e-cigarette use with smoking rates among youth in countries with strong gender bias for smoking. | Unmeasured confounding resulting in equivalence of populations on key confounders; measurement differences across populations. |
| Macro-simulation/aggregate models | N/A | Simulation models which mimic the operation of government programs and demographic processes from the top down, based on population segments moving between a limited number of states through deterministic transitions, depending on key parameters set at population level. This could be done using systems dynamics models. | Based on postulated causal association of e-cigarette use with cigarette initiation, it would be possible to construct a model to forecast likely changes in smoking rates based on changes in e-cigarette use rates and compare with observed smoking rates. The postulated causal association of e-cigarette use on aggregate state transitions can be varied to provide best fit to data. | Model misspecification (relevant confounders not included); results being only as good as the parameter estimates used and inputs included. |

*Also applies to Population-level data
